# Performative Rituals for Conception and Childbirth in England, 900–1500

**DOI:** 10.1353/bhm.2015.0076

**Published:** 2015

**Authors:** Peter Murray Jones, Lea T Olsan

**Keywords:** ritual, childbirth, conception, charms, recipes, saints, liturgy, amulets, performativity, medieval

## Abstract

This study proposes that performative rituals—that is, verbal and physical acts that reiterate prior uses—enabled medieval women and men to negotiate the dangers and difficulties of conception and childbirth. It analyzes the rituals implicated in charms, prayers, amulets, and prayer rolls and traces the circulation of such rituals within medieval English society. Manuscript records from the Anglo-Saxon period to the late Middle Ages offer evidence of the interaction of oral and written means of communicating these rituals. Certain rituals were long-lived, though variants were introduced over time that reflected changing religious attitudes and the involvement of various interested parties, including local healers, doctors, and medical practitioners, as well as monks, friars, and users of vernacular remedy books. Although many of those who recommended or provided assistance through performative rituals were males, the practices often devolved upon women themselves, and their female companions or attendants.

Other than ubiquitous death, birth holds the prime place in medieval life and thought. Birth rights secured monarchies and ownership of lands; elaborate genealogies established familial authority in the secular world. Biblical genealogies commemorated in liturgy determined the identity of Christ. The birth of Christ and the Virgin Mary’s motherhood became central [Other P-406] to Christian faith, doctrine, and religious practice. Images of Christ’s conception and nativity adorned innumerable prayer books and psalters as aids to meditation and prayer. Early ceremonies of marriage emphasized the conception of offspring under the biblical rubric of Genesis 1:28: “Be fruitful and multiply.” Baptism of infants, whose souls might be in jeopardy if they died during delivery, brought religious pressures to bear on those in attendance at births of children. Theories of conception and growth of the fetus as well as the moment when the fetus gained a human soul were subjects of debate, medical, legal, and theological.

Medical knowledge in Western Europe concerning conception and how to promote it or prevent it crystallized in texts translated into Latin from Arabic at Monte Cassino by Constantine the African in the eleventh century.^1^ This learning spread to England from the eleventh to the thirteenth centuries and was translated into English and French. From the fourteenth century gynecological texts taken from scholastic medical sources were excerpted, augmented, and widely dispersed in vernacular languages. Existing alongside such medical knowledge and writings about women’s matters was a range of ritual practices employed at crucial moments in the life experience of those who were attempting to conceive, maintain pregnancy successfully, and safely deliver healthy offspring. In England from the Anglo-Saxon period into the early modern, secular and religious rituals were recorded in a rather scattered manner in manuscripts. Some rituals were recorded because they had proved themselves effective in performance; some were preserved for future use by couples and families whose hopes were bound up in the success and failure of conception and the event of birth. In this article the terms “rituals” and “performative rituals” are used to designate verbal charms, prayers, ligatures, amulets, as well as physical gestures and sequences of actions utilizing special objects, as explained below. Rituals can refer to texts, material objects, and formal acts. The study of rituals and their communication across time and space brings to light new aspects of the medieval experiences surrounding conception and childbirth.

This article is intended to show how rituals were deployed and circulated, that is, communicated, in aid of conception and childbirth through the long Middle Ages. Its trajectory differs from that of collecting and [Other P-407] analyzing manuscript evidence of charms and amulets, as Don Skemer has thoroughly accomplished, or articulating the state of gynecological knowledge, as Monica Green has done.^2^ This article seeks, rather, to reveal medieval people in various roles involved in performing or providing rituals for conception and birth, and to bring to attention those who communicated rituals for purposes other than direct performance. The main concerns here are the channels of communication through which rituals moved and the place the rituals had in the lives of the women and men whom they affected. It is intended not to provide a chronological account of the development of ritual practices for conception and childbirth from 900 to 1500 CE but to analyze significant instances of ritual in performance, and ritual in communication.

## Charms and Performativity

Rituals, as used here, are understood to be communicative acts.^3^ The term “rituals” (or “ritual acts”) includes verbal charms, benedictions or blessings, as well as stipulated actions, such as eating or drinking words, and touching, wrapping, or tying things to the body. Rituals could be accommodated as part of medical knowledge in the Middle Ages. For example, one place to find rituals is in Monica Green’s study of the most important medieval gynecological text, the *Trotula* collection. From the inception of the *Trotula* collection, a few remedies for difficult birth relied on rituals, rather than medicinal cures.^4^ One requires having the woman eat [Other P-408] certain letters along with the *sator arepo tenet opera rotas* palindrome written in cheese or butter;^5^ another specifies a string of letters to be drunk with the milk of another woman. A third ritual employs the skin of a snake as a birthing girdle.^6^ These remedies, intended to expel a dead fetus or facilitate a delayed delivery, are found in the proto-*Trotula* versions by the eleventh century but contain elements of older childbirth rituals.^7^ Because of the wide distribution of the *Trotula*, the rituals contained within it were more accessible than most. These particular rituals, however, do not circulate verbatim outside the *Trotula*, surprisingly, given the wide distribution of this text in the Middle Ages. However, the *sator arepo* formula, which precedes the *Trotula* in time, and persists as an aid to delivery, joined with other motifs, and independently.

Verbal charms rehearse a set of instructions for performance of certain acts, and stipulate words of power to be pronounced or written on an amulet. Their purpose is to bring about a desired result. In healing they do something to someone. The prescribed rituals derive their force from their having been performed many times before. Each time a ritual is performed it draws on a tradition of previous performances, and the accumulation of prior acts creates the “illocutionary force” or power of the ritual,^8^ but each time a ritual is performed it is effective in the moment. The ritual is performed for the benefit of one particular person on one particular occasion, virtually recovering all the previous performances with different actors and different patients. Medieval healing rituals are, moreover, characterized by devices that add to their “illocutionary force” for those concerned. These might include a narrative structure to the [Other P-409] charm that is based on divine scripture, so that the ritual reenacts a legendary biblical narrative. The words of power spoken or written as part of the ritual might also be words that are spoken by Christ himself or by other sacred persons voicing the Word of God and Grace.^9^ Or the rituals might make use of what are apparently “nonsense” formulas or strings of meaningless words that may signify a vocalization or invocation of a mystery. In all such cases the ritual performance involves the creation of a new reality within which healing can take place, in which the subject of the ritual assumes a role. In doing so the beneficiary becomes subject to and persuaded by a reality different from the one in which she or he suffers. The performative healing ritual works through this shifted, constructed reality.^10^ Even in cases where no spoken words are involved, but instead prescribed actions or the wearing of an amulet, these are still healing rituals, where each performance repeats or “cites” a similar prior act, and the accumulation of prior acts creates the illocutionary force or power of the ritual. Significantly, such performative acts occur within specific social and cultural situations. To adumbrate the wider circumstances, it is necessary to pay close attention to the manuscripts in which ritual texts occur.^11^

Before considering features of the manuscripts in which ritual texts are “staged,” it is necessary to consider briefly the genre of the verbal texts. Medieval charms as a genre are frequently indistinguishable from prayers, blessings, or exorcisms within Christian tradition. If a prescribed incantation is an “oral performance to accomplish a purpose by means of [Other P-410] performative speech in a ritual context,”^12^ then it may be difficult to distinguish this incantation from other Christian rituals. What is more, charms employ motifs and formulas found in church liturgies or benedictionals, and whether we subsequently class them as charms or as something else may well depend only on the context in which they are recorded or the circumstances of their use. On the other hand, charms for conception or childbirth are also closely allied to medical recipes, sharing structural features like a heading naming the purpose, directions for performance, and efficacy statements, so that medical recipes and charms are often found intermingled in manuscripts.^13^ Charms, like both prayers and recipes, have one other feature that has a considerable bearing on issues of manuscript circulation—they are often relatively short as written textual units, and can be written in the margins of a page or in blank spaces left in manuscript books. There are rare instances, particularly in the later Middle Ages, of charms that incorporate extended devotional material, and thus become unusually lengthy, but for the most part charms for conception and childbirth are highly transferable as texts. Childbirth charms developed to significant length suggest a sense of the benefits of extended ritual performances in anticipation of the event of birth or during a delayed or painful “travailing” of birth, in the words of manuscript headings.

## Making Rituals Textual

Occasionally the manuscript record opens a window onto the process by which a verbal charm or written amulet becomes textualized. Below are two cases.

**Figure 1:**
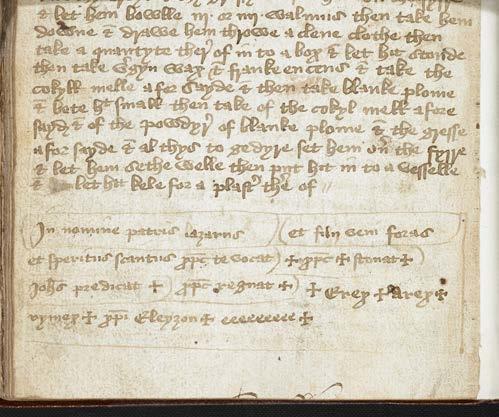
A charm to assist in delivering a child is added to the bottom margin of a manuscript leaf. Fifteenth century. London, British Library, Sloane MS 3160, fol. 129v. © The British Library Board.

First, a charm to enable delivery of a child written in London, British Library, Sloane MS 3160 bears signs of its having been recorded directly from aural memory. It was added at the bottom of a leaf (fol. 129v) by someone not fully literate in Latin or one who could speak the formula but was not accustomed to writing in Latin.^14^ Errors in the Latin (*speritus* [Other P-411] *scantus* and *stonat*) suggest how little experience the recorder of the ritual has had in writing Latin. The spoken form of the ritual is suggested by the alternation between the words of the framing formula, beginning, “In the name of the Father, Son, and Holy Spirit” and the words borrowed from the Gospel of John (11:43). Each part of the *In nomine* formula prepares for the following words of power: “In the name of the Father: Lazarus; and the Son: Come out; and the Holy Spirit: Christ calls you.” Then the threefold acclamation “+ Christ + shouts + John preaches + Christ rules +” is followed by a string of climactic vocalizations, “+ erex + arex + rymex + christi eleyzon + eeeeeeeee +.” The scribe separates the sections of the formula by brackets like verse, and inserts crosses to indicate the sanctity not only of Christ’s name but also of the unintelligible vocal expressions and the Greek words “Christ, have mercy” (see Figure 1).

In terms of performative theory, the last words belong to the constructed reality, the transcendent realm in which the woman in labor becomes now situated. Oral performance seems necessary for the success of this aid to delivery, because of its complex utilization of voices and sound patterns and its direct recourse to the power of Christ’s spoken words.^15^ It engages prior uses of the Lazarus story for delivery of the child, that appears in an Anglo-Saxon ritual discussed below. The infant/ Lazarus identity belongs to the common oral-aural world of the speaker and hearer of this ritual.^16^ Many other late medieval occurrences of this motif reinforce its performative effect. The historicity of its prior uses determines its effect, while the performance works uniquely in the present situation to bring on delivery of a child.

**Figure 2:**
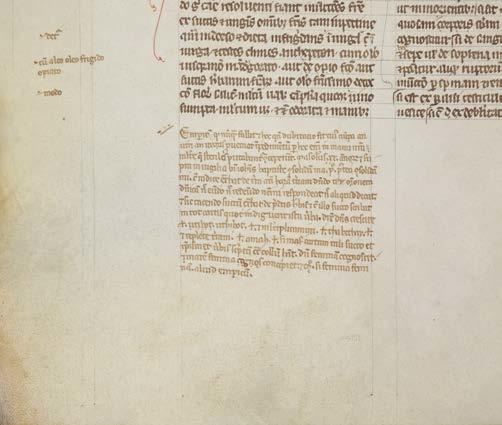
The *“empericum* that never fails” in the margin of the *Compendium* of Gilbertus Anglicus. The instructions are for making and applying an amulet for conception. Thirteenth century. Cambridge, Pembroke College, MS 169, p. 449.

Second, an early manuscript of the widely known physician and medical author called Gilbertus Anglicus (Gilbert the Englishman) contains the marginal record of what came to be known as Gilbertus’s “ *empericum* that never fails,” according to its opening line. The instructions for making and applying an amulet for conception appear in the bottom margin of Cambridge, Pembroke MS 169, p. 449, along with clear indications of where it should be added into the main text (see Figure 2).

The text claims to be a firsthand account, either by Gilbertus or the source of the note (“By this method by our hand many who were thought [Other P-412] to be sterile have conceived”).^17^ The prescription begins with instructions for a ritual to be carried out by a man of twenty years or more, evidently the prospective father. He is to gather herbs and use their juices to write an amulet to be worn around either the man’s or the woman’s neck during intercourse, depending on the sex of the child desired. This prescription appears along with other medical information and remedies in the margins of the manuscript. Some added material seems to have been gathered from outside informers. Some are identified, such as, “Sarracenus et suriani” (p. 211), “Quidam miles” (p. 75), and “Quidam docuit me” (p. 73), while other marginal additions are quotations from the writings of ancient authorities, for example, “Ptolomeus dicit” (p. 174).^18^ The *empericum* for conception, like other additions, subsequently appears inside the main body of Gilbertus’s text in manuscripts through the fourteenth and fifteenth centuries and into the printed edition of 1510. The very word *empericum* in the famous physician’s book is an indication that it was considered a practical remedy, not based in humoral theory, but trusted because it had been proven to work by experience alone. If this addition, like others identified by Michael McVaugh, was Gilbertus’s own and intended to be added to his *Compendium*, then it appears that the physician is here appropriating a practice from outside medical theoretical knowledge for inclusion in his seven-book compendium of practical medicine. Once inside the main body of Gilbertus’s book, this conception amulet acquires a new circulation, among those who could afford to[Other P-413]

[Other P-414]

commission or buy a copy of the impressive medical text. Once a ritual became fixed within a medical compendium or a remedy book collection of recipes, either it could be returned to living practice in oral-aural performance by subsequent selection and use or, alternatively, it might continue to exist in a textual form without being performed.

## Persistence of Motifs: The Sequence of Holy Mothers

Some ritual motifs survived over centuries. The advantage of a long-term perspective is that differences of expression of a single motif can be identified in changing contexts. Within medieval Christianity, one of the longest surviving motifs is the sequence of holy mothers (sometimes referred to as the *peperit* charm).^19^ The earliest known occurrence in England of the sequence of holy mothers appears in an eleventh-century Anglo-Saxon manuscript containing Lenten sermons, including dialogues between the soul and body.^20^ A charm for a pregnant woman near term is the first of four charms written in the space available within the sermon texts. The supplementary status of the charm texts suggests that the charms coincided with the clerical purposes of the main texts. It is likely that they were joined with these sermons because they were employed by churchmen in pastoral care. The charm exemplifies four long-lasting elements in medieval childbirth charms: (1) the sequence of mothers; (2) an adjuration to the infant (“ut exeas”) and exorcism; (3) the account of Lazarus’s resurrection according to John with its climactic words, “Lazarus, come forth!”; and (4) directions for making an amulet to be tied to the right foot of the woman.

The Virgin Mary gave birth to Christ
Sterile Elizabeth gave birth to John the Baptist
I adjure you, infant, whether you are male or female,
through the Father and Son and Holy spirit that you go out and depart
in addition may you [demon] not harm this one [the mother?]
nor cause that one [the child] to be senseless amen.
The Lord, seeing Lazarus’s sisters weeping, shed tears at the tomb in the presence of the Jews and shouted:
“Lazarus come out” And he came out, with his hands and feet bound, he who had been four days dead. Write this on virgin wax
and bind on her right foot^21^

[Other P-415]

The amulet is the most interesting part of this ritual. It can hardly be labeled as antireligious, in the light of the religious, especially penitential, character of the manuscript in which it is written and the full incorporation into the ritual of the Gospel material as an aid to delivery. The use of wax that has never been worked (“næfre ne com to nanen wyrce”) suggests a sacramental quality based on its purity. Although there are no directions to speak or chant the words over the woman, the liturgical correspondences make it most likely that they were indeed spoken over her. That the whole charm is written in wax and tied to the woman’s right foot is a significant performative act directly impacting her body. Moreover, it seems likely that this happens while she is in the throes of labor. The written amulet allows the quoted words to be affixed to the woman before or during labor. The words supply a frame for events and, being affixed to her body, mediate her experience through touch and sight in addition to her having heard them.^22^

The holy mothers or Tree of Jesse ritual for childbirth enjoyed great popularity on the European continent.^23^ In England, written evidence [Other P-416] of the ritual after the eleventh century seems to be lacking until the second half of the thirteenth century. Tony Hunt’s work shows that a short form appears in Latin remedies associated with the Anglo-Norman *Lettre d’Hippocrate*. In addition to Mary and Elizabeth found in the Anglo-Saxon manuscript, Anna, mother of Mary, and Celina, mother of Remigius, appear. The writing required for the amulet placed on the belly also includes the *Sator arepo* palindrome. Another birth amulet for the right foot requires the beginning of Psalm 49, “God is called the lord of gods and he called the earth.”^24^ The Latin holy mothers ritual appears in Hunt’s thirteenth-century manuscripts most often introduced by an Anglo-Norman rubric. This rubric directs that if a priest or cleric is present, he should read the written text over the head of the woman in labor after which the script (*bref, brevet*) is to be put to her belly.^25^ This expression of the motif envisages an active performative role in the ritual for the cleric. Added to the threefold sequence (Anna, Mary, Elizabeth) are often words asking for mediation through the merits of the Virgin or saints. The French versions tend to be intensely prayerful and express devotions to named saints.^26^

By contrast simpler Latin forms of the genealogical ritual consisting of three holy mothers and a *sator arepo* palindrome are found in fourteenth-and fifteenth-century manuscripts, and are more suggestive of a medical emergency. Two examples are found together in one manuscript: a short version of the holy mothers motif together with the *sator arepo* is used to aid in a live birth. This ritual follows a medical warning that bloodletting in pregnant women is perilous. The second version begins “*Anna* [for Hannah] *peperit Samuel*,” and is to bring forth a dead child. The practitioner makes the sign of the cross as he steps on the threshold of the house, then recites the genealogical charm, a *pater noster* and *ave maria* each three times. The rituals are meant to protect the life of the mother, yet the charm text asks that she, like Hanna, Elizabeth, and Maria, produce, [Other P-417] then exhorts the child—presumed dead in the heading—to come out for baptism (“veni foras ad baptismum”). Thus, the traditional words of the formula and the promise of baptism are not changed in this expression of the motif by the supposition of a dead rather than a living child.^27^

In the fifteenth century, the holy mothers motif is expressed in two different forms, which were both widely disseminated in manuscript remedy books. Remedy books are collections of medicinal recipes, prognostications, and charms, often written in a blend of three languages, Latin, Anglo-Norman (French), and Middle English. It is seldom clear who commissioned, wrote, or read these manuscripts, but most probably they were used for practical purposes in the context of literate households and religious communities.^28^ One remedy book version consists of a single long amuletic text, which contains a string of liturgical motifs including the sequence of holy mothers, twice. The manuscripts in which this ritual appears have been called the “Leechcraft” remedy books.^29^ No other ritual for childbirth is found in this group. The charm (*Carmen*) or *writ* is to be tied to the right thigh of the woman in labor. Though the title may appear in English, the text itself is entirely in Latin. It employs two genealogical motifs, one beginning with Mary and the other with Anna, plus a series of widely known liturgical chants. For example, the chant [Other P-418] beginning “deus ultionum” (Deus ultionum dominus deus ultionum / God of vengeance, Lord God of vengeance; Psalm 93:1) appears with musical notation in manuscripts dating from the twelfth century; it belongs to the Offices for Fridays.^30^ But in this charm, it is transformed into a direct appeal to the God of vengeance and power on behalf of the woman, “your servant,” whose name would have been supplied by the speaker of the ritual or writer of the amulet. One reason the lengthy verbal ritual may have been meant to be written on an amulet, rather than spoken, may have been that a male religious or medical authority could write it and give it to the female or females who were attending during the actual delivery, who would attach it. The very length of this ritual might suggest a long chant on behalf of the woman, but in the Leechcraft remedy books, it is prescribed as an amulet.^31^ This would not prevent the recitation of its paraliturgical texts by the parturient woman or on her behalf by her companions. As an amulet, this long form is distantly related to prayer rolls employed as birth girdles, discussed below.

The other well-known fifteenth-century remedy book version is short and appears as one of several alternative verbal rituals for childbirth. This group of remedy books, best known through the manuscript copied by the Yorkshire gentleman Robert Thornton, supplies a set of much shorter performative rituals for “travayling of child.”^32^ The holy mothers charm names “Beata Anna” who gave birth (“genuit”) to “Sanctam Mariam,” but not using the *peperit* formula. Rather, it emphasizes that this birth is essential to salvation according to the creed. It focuses on a spoken ritual that could be voiced by any Christian comfortable with speaking Latin words, including the parturient woman, who is named as the recipient [Other P-419] of the intercessions of Mary.^33^ The other short verbal rituals for childbirth found in this series in the Thornton group remedy books survive from late antique or early medieval sources. The Latin *Arcus* flourishes in charms of the fifteenth century.^34^ Another charm in the series takes a fragment from Virgil’s *Aeneid* XI.1, referring to Aurora rising out of the waters of “Oceanum.”^35^ The Athanasian Creed denoted by its first words (“quicumque vult”) also occurs as one of the series.^36^ The appearance of the holy mothers associated with other short rituals surfacing in fifteenth century manuscripts belonging to the Thornton group implies that the householder can choose between the holy mothers motif and others, secular or religious, while the single long ritual found in the contemporary Leechcraft group demonstrates that a long-lived childbirth ritual can be “staged” for devotional performances or amuletic use.

## Medical Prescriptions

The most widely circulated medical author in England in the later Middle Ages was the surgeon John Arderne (ca. 1307–ca. 1380), who wrote in Latin but was translated four times into Middle English. His *Liber receptorum medicinalium* (Book of medicines) is a disorganized collection of advice and remedies, mostly surgical, which has survived in nearly forty manuscripts. Included is one charm for speeding delivery of a child:[Other P-420]

To make a woman deliver a child quickly after a long labor. Bind this charm below the knee of the woman in labor while saying the Lord’s Prayer and the Ave Maria. + Just as we believe that the word + is made + flesh + and that the Virgin Mary bore the infant Jesus as both true God and man, so with Mary interceding and our Lord Jesus Christ bidding, may you successfully give birth to the child you bear in your womb. When Christ was born his mother suffered no pain. Once more Christ is born as both God and man + Christ calls you child + come out + come out + Christ conquers + Christ rules + Christ is lord + may Christ defend you from every evil amen + Michael + Gabriel + Raphael + come to his aid.^37^

This written amulet bound to the body relies on the invocation of the word made flesh from the Angelus devotion, the Virgin birth, the *exi foras* motif, the benediction *Christus vincit*, and the assistance of the archangels. While many of these motifs are also found in the rituals associated with the sequence of the holy mothers, only the Virgin’s giving birth is called on here. Arderne drew on local informants as well as other medical writings as sources for his prescriptions, but in this case his amulet has a strongly liturgical and intercessional character.^38^

English medical authors like Arderne represent an important medium of transmission for rituals to do with childbirth, because they were acknowledged sources of authority for healing. However their impact on medical practices concerning conception and childbirth is harder to assess. We can bring to bear on this question of impact a surviving manuscript book assembled and partly written by one medical practitioner, who left to his heirs and successors a kind of commonplace book of the [Other P-421] remedies he made use of himself and recommended to others.^39^ Thomas Fayreford practiced at Bridgewater in Somerset and Tiverton in Devon in the first half of the fifteenth century. His practice was remote from the book-producing centers of London, Oxford, and Cambridge. He drew on scholastic medical writings, remedy books, and local informants as sources for his prescriptions. Thus he refers to a “Master Lowes” as his source for prescribing herbs and prayers to be said over a woman who cannot feel her fetus move and is troubled by illness in pregnancy. Among many amulets and rituals recorded in his book is one for conception. Fayreford’s amulet to make a woman conceive should be written on lead. He drew in his book a diagram of two embedded rectangles with a ladder across it diagonally with the following letters inscribed: “os. T. acori. sa: t. p + υ[upsilon].ii. N.d..”^40^ This diagram is in effect a blueprint for making the amulet concerned. By comparison with the remedy books or other medical literature of the period, where the relation to practice remains uncertain, we encounter here a text that is prescribing the making of a ritual object to the reader of the book, and that reader is assumed to be reading for an active purpose—perhaps it may be Fayreford himself in the course of his own medical practice, or perhaps later owners of the book.

He also prescribes three other amuletic rituals to speed up a difficult delivery. These are all described in Fayreford’s manuscript as *experimenta*, that is, procedures, rituals in this case, that have been found by experience to be effective.^41^ The first prescribes writing verses on paper or new parchment to be bound to the woman’s thigh. The verses include two lines making a typological comparison of Eve and the serpent with the Virgin conceiving through the ear (“vipera vim perdit sine vi pariente recedit / dum sacra virgo dominum aure concepit”)^42^ and two lines from the [Other P-422] beginning of the tenth book of Virgil’s *Aeneid* (“Panditur in terra [*sic*: for interea]. domus omnipotentis olimpi / concilium que vocat omni [*sic*] pater ac hominum rex”). This amulet, Fayreford claims, has been tested on many.^43^ A second ritual for speeding birth requires lines from Psalm 115 to be written on new parchment (“dirupisti vincula mea tibi sacrificabo hostiam laudis et nomen domini invocabo”), then read over the head of the woman, tied with a seal and bound to the large joint of her finger with a red silk thread. The third ritual must be written in three sections on communion wafers, and invokes Lazarus (who is summoned out of his tomb) in the name of Father, Son, and Holy Ghost.^44^ The woman then consumes one wafer at a time, and if any wafers are left uneaten they must be burned. It rarely happens that a third wafer is required, Fayreford tells us. Although Fayreford was not a cleric, his childbirth amulets all require the writing (and in one case reading) of Latin religious texts, as well as extracts from Virgil, a feature we have met already in the Leechcraft remedy books. The third amulet assumes that communion wafers, the most important sacramental objects of all, can be obtained to help deliver the woman in childbirth through the offices of a priest. Male agents, clerical or lay, play the leading roles in these ritual interventions in childbirth.^45^

Other kinds of practitioner were also happy to prescribe childbirth rituals. Mendicant friars seem frequently to have engaged in medical practice in late medieval England. A number of cases in which they were active practitioners are recorded in the *Tabula medicine*, a handbook of remedies in disease order written in Latin by a consortium of English friars between 1416 and 1425. They displayed a keen interest, perhaps surprisingly, in gynecological and obstetric matters in particular. Among the many headings to do with women and their ailments we find, under the heading for [Other P-423] “Partus” (birth), an example of the sequence of holy mothers, followed there by the *sator arepo* palindrome. The prescribed amulet is to be bound to the belly of the woman, and is essentially the same as that circulating ca. 1300.^46^ The *Tabula medicine* enjoyed a circulation among the mendicants for whom it was written, but also became popular after midcentury with physicians in England. Presumably the friars who made use of the book were expecting on occasion to have to perform the ritual over a woman giving birth, or were asked by lay people to supply them with such a help to delivery. It is clear that mendicants and physicians, many of the latter being in clerical orders, had no inhibitions in prescribing childbirth rituals and may have been actively involved in the performance of such rituals.

## Prayer Rolls as Birth Girdles

Don Skemer has thoroughly surveyed the contents and production of a wide range of written amulets and their exemplars, including prayer rolls for women.^47^ One type of written amulet, late medieval prayer rolls, could be used as birth girdles. The use of elaborate rolls involved ritual practices neither confined to women nor disapproved of by churchmen. A soldier, a sick man, any sinner facing the spiritual danger of imminent death might seek the benefits offered by such a roll. But their acquisition belonged among the customary practices surrounding childbirth—infant baptism, lying-in, and churching of postpartum women—especially in wealthier circles during the late medieval period. From the perspective of the women who employed them, the husbands who could commission them, and the canons or other religious men (we know of no women) who designed and wrote them, they displayed a fervent, orthodox, if sometimes idiosyncratic, piety. One such roll now in the Morgan Library in New York [Other P-424] was made by a canon of the Abbey of Coverham in Yorkshire;^48^ rolls or “girdles” are known to have been kept by English religious institutions for lending out to pregnant women; a monk was rewarded for delivering “our Lady gyrdelle” to Elizabeth, queen of Henry Tudor, six weeks before the birth of her eleventh child in 1502.^49^

During the fifteenth century, the veneration of St. Margaret of Antioch, Saints Quiricus and Julitta, the Virgin, and the instruments of the passion of Christ inspired the production of amuletic rolls particularly suited to a devout woman nearing parturition.^50^ Two manuscript rolls in the Well-come Library contain the life of St. Margaret written in French.^51^ In both Anglo-Norman and English versions of Margaret’s Life, the saint prays that any house containing a copy of her passion (i.e., a manuscript of the Life) may have no deformed children in it, and that if a woman in labor calls on her she may be safely delivered of a live and healthy child.^52^ If the book is read aloud to her it will serve the same purpose. The book itself thus becomes a protective amulet, whether as a physical presence or as the exemplar for a telling aloud of Margaret’s Passion.^53^ There are surviving texts from France of Margaret’s life written on scrolls or folded up as *brefs* that were clearly attached to the pregnant woman.^54^[Other P-425]

Few prayer rolls that might be used as birth girdles have survived from medieval England, perhaps because they were liable to wear out or were destroyed in the Reformation.^55^ To women, they offered protection primarily from the two great dangers of childbirth—sudden death while in a state of sin and the death of the unbaptized child.^56^ If an infant died before being baptized, there was no salvation of her soul. On Wellcome Library, MS 632, the guarantee of protection from sudden death in battle, from fire, water, wind on sea or land, pestilence, and other troubles like robbery, was promised in a legendary epistle delivered from heaven to an earthly magnate.^57^ At the end of the list, appears the promise specifically for a woman: “And yf a woman travell wyth chylde gyrdes thys mesure abowte hyr wombe and she shall be safe delyvyrd wythowte parelle and the chylde shall have crystendome and the mother puryfycatyon.”^58^ The woman who girds herself in the measure of the length of Christ will deliver safely; moreover, the child will live to be baptized and she to be purified at the church after the birth in due course.

While a “heavenly letter” brings promises of protection from above, intercessory prayers, such as those to Saints Quiricus and Julitta, also posit a vertical communication. Both function as performative rituals reconstructing the self of the devotee and expanding the dimensions of her world. Prayer rolls and birth girdles make another impact as material objects. First, prayer rolls might function as amulets, as above, by being placed on the body. Second, the visual images painted on the rolls were the focus of ritual gazing, “devoutly looking upon” the images, as well as veneration through touching or kissing.^59^ Meditating on the wounds [Other P-426] of Christ, for example, entails rehearsing the number of the drops of blood spilled on behalf of Christians; prayers to the Wound in Christ’s side stimulate thoughts about the painful torments Christ suffered and the refuge from pain offered by the heart wound, known as the “well of salvation.” The theological conjunction of Christ’s pain with the liberation from sin and death might resonate with a woman about to undergo the pains of labor and the threat of death. Third, rolls contained instructions, in English, for the ritual uses of their contents, including prayers for recitation in Latin and images, combined with meditations on the measure of Christ’s height, the wound, and the drops of blood. Fourth, the narrow width of some rolls represented the belt shape of the Virgin’s own girdle, a relic that according to legend she dropped to St. Thomas at her Assumption.^60^ This construction allowed the whole length of the roll or specific images to be laid over a woman’s pregnant belly, as directed in the heavenly letter, or to be girded about her person. Performance of the rituals guaranteed to those praying with a roll that salvation of the soul was certain. The proximity of the object alone, its talismanic value, or having looked at it on the day preserved one from dying suddenly in a state of sin. For a woman “travailing of child,” its purpose was to preserve her and her child through the dangers of birth. In one case, the *sator arepo* and “sancta anna peperit beatissimam virginem mariam” are prescribed as an amulet for a quick and painless delivery.^61^ Such a charm inscribed on a birthing girdle as part of a complex meditative and visual program illustrates a marked change in media from oral performances of charms depending entirely on the power of spoken words and broad-ranging ritual actions, as seen below.

## Rituals Performed by Women and Clerics: Anglo-Saxon Evidence

Among the oldest materials available to us for England are a set of Anglo-Saxon rituals for problems surrounding the conception and healthy gestation of a child.^62^ These spoken words are metrical and vernacular. They [Other P-427] seem to draw on local customs, but are recorded in a monastic collection of medical remedies (in London, British Library, Harley MS 585). The set begins with Old English vernacular, poetic formulas, “for a woman who cannot nourish her child,” that is to say, she cannot successfully bring her child to term and safely deliver it. The vulnerable woman must recite the verses as she steps over a grave, perhaps that of a previously lost child. The poetic lines of the first section are mirrored in the section immediately following, which ensures the healthy development of the unborn fetus. The self-reference of the woman speaker is extraordinary. In the first two parts, she performs the two stepping rituals in different places as she speaks the words. In the third ritual, the woman goes to a church to announce her pregnancy. The lines to be spoken are presented metrically with translations alongside.

þis me to bote þære laþan lætbyrde; This be a help to me for the hateful late birth
þis me to bote þære swæran swærtbyrde; This be a help to me for the black birth
þis me to bote þære laþan lambyrde. This be a help to me for that hateful slow birth

When a woman knows that she is pregnant, she can seek to ensure good progress by repeating this charm while she steps over her husband lying in bed:

Up ic gonge, ofer þe stæppe       Up may I go, over you may I step
mid cwican cilde, nalæs mid cwe[l]endum, With a living child, not with a dying one
mid fulborenum, nalæs mid fægan’. With a full-term one, not with a failing one

When a pregnant woman is certain that the child is living, she is to go to the church and, standing before the altar, declaim the following:

Criste ic sæd þis gecyþed. Christ I said this [child] is revealed.^63^

After these rituals, there are two more birth-related metrical recitations and ritual acts written into the manuscript. One gives directions for a woman who has lost a child and buried it: the grieving woman takes from the grave a bit of the soil, which she wraps in black wool and sells to a [Other P-428] merchant, saying, “I sell it; you buy it / this black wool and this grain of sorrow.”^64^ The second seems to be for safely bringing an infant to term or for ensuring lactation after a child is born.^65^ This last ritual requires an extended sequence of actions. The woman is to take milk from a cow of one color and sip it from her hand and spit it out into a flowing stream. Then she must take water from the running stream with the same hand, a mouthful, and swallow it, and recite rather heroic verses, “Everywhere I have carried the glorious well-formed son. / By means of this glorious, strong food / I will keep him for myself and go home.” The instructions state that as she walks to and from the stream, she should not look behind her, and that after leaving the stream, she must go to a house, not her own, and eat something.^66^

These rituals are unique in the medieval material first because they clearly rely on verses and acts drawn from local vernacular sources and second, because in no other medieval performative rituals are the voice and actions of an individual woman who will be performing these rituals so clearly represented. Her acts and words reiterate traditions that have acquired an authority in an oral-aural world of what a woman can do to enhance her chances of successful conception and gestation and the birth of a healthy baby. Moreover, the focus in these Anglo-Saxon rituals is not on the last states of labor and delivery, rather on successful generation and gestation. The traditionality or historicity of her words and rituals are in large part self-directed. These performative acts impact her motherhood directly, creating the framework for success, where failure is a recognized likelihood or even a past reality. The verbal formulas name the worst fears, while performance of the stepping, selling, and sipping rituals persuades [Other P-429] through words and bodily acts that something not only can be done, but has been done that creates a new reality. The last ritual of taking the milk, going to the stream silently, and taking food at a friend’s house suggests that other people, most likely women, were aware and supportive of that ritual, as the presence of a sleeping husband is essential in the second set of stepping verses.

The Anglo-Saxon oral tradition of vernacular metrical charms to relieve problems associated with childbirth disappears from view later in medieval England. The metrical tradition may have been dying out during the Anglo-Saxon period. However, in Anglo-Saxon manuscripts, there is ample evidence of priests or other religious being involved in the preparation of medicinal remedies.^67^ Clerical involvement in rituals to bring about conception and a successful delivery is found in a prayer charm or benediction recorded in the margins of an English translation of Bede’s *Ecclesiastical History of the English People*. The main text of the manuscript was copied by the middle of the eleventh century. The margins were filled within a few decades afterward with a rich collection of liturgical and homiletic pieces and a few charms and prayers, including a Latin benediction asking for a woman’s fertility and painless childbirth.^68^ The benediction begins by addressing the Trinity as creator (“Creator et sanctificator pater et filius et spiritus sanctus”). *Sator arepo* is inserted where a reference to the Trinity might be expected: “precamur te domine clematissime pater ut elemosina ista fiat misericordia tua ut accepta sit tibi pro anima famuli tui ut sit benedictio tua super omnia dona ista per [Other P-430] + Sator . arepo . tenet . opera . rotas.” (we pray to thee, Lord most merciful Father, that through these alms your mercy be done, that [they] be acceptable to Thee for the soul of your servant, that your blessing be upon all these gifts through + Sator . arepo . tenet . opera . rotas). This Anglo-Saxon marital benediction for a woman’s fertility and painless childbirth was evidently intended for performance by a cleric.^69^ It concerns human procreation as the principal marital concern and leaves room to insert the name of the woman for whom the ability to conceive and attain successful, painless delivery of children it seeks. It appears to be an unofficial church benediction to be spoken by an authoritative church figure.^70^ The Anglo-Saxon woman named in the ritual becomes part of a communicative discourse that includes her husband, referenced in the first half of the prayer (“famuli tui”), the officiating clergy, God, and those in attendance. The cleric frames human procreation within the divine plan and directly dispenses prayers including the venerable words of the *sator arepo* for fertility and successful birth.

## Conclusions

This article addresses the communication of performative rituals for conception and childbirth. The project encompasses the questions of how and by whom such rituals were performed, the nature or content of such rituals, and the ways in which knowledge of the rituals was circulated in manuscripts. Verbal rituals and amulets moved back and forth from oral-aural culture to manuscript, and from manuscript to manuscript or new performance. In contrast, the copying of ritual texts was on rare occasions a matter of antiquarian interest (as with William Worcester, n. 45).

Ritual treatments for conception were relatively rare. In the Anglo-Saxon period clerics with medical concerns collected a ritual (to be administered by herself) for the woman anxious to conceive a child and safely bring it to term. By the thirteenth century written amulets were in circulation, prescribed by learned doctors like Gilbertus Anglicus for couples who sought help. Although these amulets could require an elaborate process of construction, perhaps by one or both of the couple, the [Other P-431] prescriptions themselves have the air of authoritative medical knowledge communicated to medical patients.

Compared to the rare examples of ritual therapies for conception, rituals for women confronting the throes of labor, both to speed delivery and cause delivery of a dead fetus, are frequently found. They consist of chanted, spoken, or written words and sometimes they are meant to be “read over” a woman, then applied to her body. The variety in verbal content of the motifs of these rituals is limited. From the Anglo-Saxon period to the early modern, the same verbal formulas appear repeatedly, occasionally alone but most often combined—for example, *Sator arepo*, the Maria *peperit* (and its variations), *exi foras*—both as spoken rituals and as rituals written and tied to or laid upon the body of the woman in labor. The widely known charm motif of the sequence of the holy mothers took different forms over time and within distinct communities of readers.

The ecclesiastical dissemination of the Latin charms for childbirth can hardly be doubted, given both the liturgical content of the motifs and the witness of the manuscript records in which they first appear.^71^ However, it is equally clear that from the fourteenth century at the latest, medical practitioners were supplying rituals for conception as well as childbirth to their patients, presumably in person. At the same time anonymous recipe books and medical miscellanies were available to any household of sufficient wealth to have a copy made, and both kinds of manuscript contained ritual treatments for women at the point of delivery. In the high and late Middle Ages, most of those present during a birth were women, but men were close by, interested, and occasionally called on for special duties.^72^ A priest might be present, who had been sent for, as the French instructions indicate, or a medical practitioner, especially in cases of emergency. These men might offer a ritual in the form of a charm or amulet.

During the later Middle Ages, we find a growing expression of personal piety, and rituals involving laying the words on her belly or chest in the form of birth girdles. By the end of the fifteenth century, personal prayers and petitions for the intercession of Christ, the Virgin, and helping saints [Other P-432] like Margaret and Anne express the hopes of direct intervention for successful conception as well as birth.^73^

Most medieval rituals employed the language of ecclesiastical Latin and relied heavily on traditional biblical and apocryphal materials, even when most of the surrounding texts were written in the vernaculars of English or French. These religious rituals also preserve formulaic traditions of incorporating powerful names, in exotic languages like Hebrew and Latin, a mark of their performative character as well as their traditional framework. The staging of these traditions changes over the centuries. But exceptions to the ecclesiastical contents should also command attention. While John Arderne offers the church prayers for the woman in labor, Fayreford advocates a diagram, a ladder with letters, in his amulet for conception; moreover, he will offer lines from Virgil to speed delivery as an alternative to Christian rituals. The presence of these performative rituals in manuscripts suggests that the expectation and hazard of childbearing was a matter of crucial importance not only to women themselves but to a wide range of males who recorded these rituals and may have been involved in their performance.[Other P-433]

## Footnotes

1
See Constantine the African, Liber de coitu: El tratado de andrología, estudio y edicion critica, ed. and trans. (into Spanish) CartelleEnrique Montello (Santiago de Compostela: Universidad de Santiago de Compostela, 1983); perhaps more influential still were the relevant sections of Constantine’s translations of the *Viaticum* and *Theorica Pantegni*.

2
SkemerDon C, Binding Words: Textual Amulets in the Middle Ages (University Park: Pennsylvania State University Press, 2006); GreenMonica H, “From ‘Diseases of Women’ to ‘Secrets of Women’: The Transformation of Gynecological Literature in the Later Middle Ages,” J. Mediev. Early Mod. Stud. 30 (2000): 5–39; GreenMonica H, ed. and transl., The “Trotula”: A Medieval Compendium of Women’s Medicine (Philadelphia: University of Pennsylvania Press, 2001); GreenMonica HMooneyLinne, “The Sickness of Women,” in Sex, Aging, and Death in a Medieval Medical Compendium: Trinity College Cambridge MS R.14.52, Its Texts, Language, and Scribe, Medieval & Renaissance Texts and Studies 292, 2 vols., ed. TavorminaM Teresa (Tempe: Arizona Center for Medieval and Renaissance Studies, 2006), 2 1: 455–568; GreenMonica H, Making Women’s Medicine Masculine: The Rise of Male Authority in Pre-modern Gynaecology (Oxford: Oxford University Press, 2008); a bibliography on medieval women, gender and medicine up to 2009 is to be found at http://www.sciencia.cat/biblioteca/documents/Green_CumulativeBib_Feb2010.pdf (accessed 8 2, 2014).

3
LaidlawJamesHumphreyCaroline, “Action,” in Theorizing Rituals: Issues, Topics, Approaches, Concepts, 2 vols., ed. KreinathJensSnoekJanStausbergMichael (Leiden: Brill, 2006), 1:265–83. Laidlaw and Humphrey use the term “coercive communication,” in contrast to their own definition of ritualized action as “non-intentional, stipulated, elemental or archetypal” (quotation on 278).

4
Green, “Trotula” (n. 2), nos. 98, 100, and 102 The sources of these remedies are not known, according to GreenMonica H, “The Development of the *Trotula*,” essay 5 in Women’s Healthcare in the Medieval West: Texts and Contexts (Aldershot: Ashgate Variorum, 1996), 187–88.

5 “Vel scribantur hec nomina in caseo uel butyro: + sa. e. op. ab. z. po. c. zy. e. pe. pa. pu. c. ac. sator arepo tenet os pera rotas.” Green, *“Trotula”* (n. 2), 100, with notes of variants. The opening sign of the cross suggests that the formula was considered to be Christian. The *sator arepo* formula appears in Christian contexts early in the medieval period (see n. 68 below).

6 “cingatur mulier de spolio serpentis de quo serpens exiuit” (Likewise, let the woman be girded with a snake’s skin from which the snake has emerged). Green, *“Trotula”* (n. 2), 102, 103.

7
EgertFerdinand Paul, Gynäkologische Fragmente aus dem frühen Mittelalter nach einer Petersburger Handschrift aus dem VIII.-IX. Jahrhundert, Abhandlungen zur Geschichte der Medizin und der Naturwissenschaften, Heft 11 (Berlin: Emil Ebering, 1936), 20.

8
“Illocutionary force” is the term given to this phenomenon by AustinJ. L, How to Do Things with Words. The William James Lectures delivered at Harvard University in 1955 (Oxford: Clarendon Press, 1975 For its application to injurious speech, see ButlerJudith, “Burning Acts-Injurious Speech,” in Performativity and Performance, ed. ParkerAndrewSedgwickEve Kosofsky (New York: Routledge, 1995), 197–227. Butler’s explanation of “the accumulating and dissimulating historicity of force” (205) applies equally well to words and actions employed in healing rituals.

9
On Grace as an ingredient in medical remedies, see MilnerMatthew, “The Physics of Holy Oats: Vernacular Knowledge, Qualities and Remedy in Fifteenth-Century England,” J. Mediev. Early Mod. Stud. 43 (2013): 219–45.

10
For early medical thinking on how incantations may reconstruct a patient’s mental world, see the Latin translation of Qustā ibn Lūqā’s De incantatione adiuratione et colli suspensione in WilcoxJudithRiddleJohn M, “Qustā ibn Lūqā’s Physical Ligatures and the Recognition of the Placebo Effect,” Mediev. Encount. 1 (1995):1–50 (esp. 33, lines 58–64, line 73, translated p. 42) and Urso’s aphorism 39 and gloss in Maaike Van der Lugt, “The Learned Physician as a Charismatic Healer: Urso of Salerno (Flourished End of Twelfth Century) on Incantations in Medicine, Magic, and Religion,” Bull. Hist. Med. 87 (2013): 307–46, Appendix 2 (335–46). For later theories regarding the power of incantations, see DelaurentiBéatrice, La puissance des mots “virtus verborum”: Débats doctrinaux sur le pouvoir des incantations au Moyen Âge (Paris: Les Éditions du Cerf, 2007) and FangerClaire, “Things Done Wisely by a Wise Enchanter: Negotiating the Power of Words in the Thirteenth Century,” Esoterica 1 (1999): 97–132, http://www.esoteric.msu.edu/Fanger.html, (accessed 8 2, 2014).

11
On performative texts in medieval manuscript contexts, see StuartHeatherWallaF, “Die Überlieferung der Mittelalterlichen Segen,” Zeitschrift für deutsches Altertum und deutsche Literatur 116 (1987): 53–79 and especially HaeseliChrista M, Magische Performativität: Althochdeutsche Zaubersprüche in ihrem Überlieferungskontext, Philologie der Kultur 4 (Würzburg: Königshausen and Neumann, 2011), 9–27, 61–86, and 103–19.

12
OlsanLea, “The Inscription of Charms in Anglo-Saxon Manuscripts,” Oral Tradition 14 (1999): 401–19, quotation on 403.

13
OlsanLea T, “Latin Charms of Medieval England: Verbal Healing in a Christian Oral Tradition,” Oral Tradition 7 (1992): 116–42; recipes are discussed in CarrollRuth, “The Middle English Recipe as a Text-Type,” Neuphilologische Mitteilungen 100 (1999): 27–42. See also HuntTony, Popular Medicine in Thirteenth-Century England: Introduction and Texts (Cambridge: D.S. Brewer, 1990); JonesClaire, “Formula and Formulation: Efficacy Phrases in Medieval English Medical Manuscripts,” Neuphilologische Mitteilungen 99 (1998): 199–210.

14 The scribe writes English in a Warwickshire dialect. See the manuscript description in “Manuscripts of the West Midlands” online at www.hrionline.ac.uk/mwm/browse?type=ms&id=83 (accessed August 2, 2014).

15
Olsan, “Latin Charms” (n. 13) 121.

16
FoleyJohn Miles, Immanent Art: From Structure to Meaning in Traditional Oral Epic (Bloomington: Indiana University Press, 1991), 6–8, 42–60, called this phenomenon “traditional referentiality.” For eleventh-century charms featuring Lazarus, see HeimR, Incantamenta magica Graeca Latina (Leipzig: Teubner, 1892), 550 and Oxford, Bodley MS Junius 85, fol. 17.

17
Translated, it reads, “A remedy that never fails and this will succeed when there is doubt about whose fault it may be, whether the impediment lies with the husband or the wife. Through this treatment by our hand, many who were thought to be sterile conceived: On the eve of the feast of the blessed John the Baptist before the third hour, have a man twenty years of more pull from the earth comfrey first and, afterwards, daisy with its root, while saying the Lord’s Prayer three times. And neither going nor coming should they [sic] respond to anyone or say anything, and thus keeping silence let him extract the juice from the forementioned plants and with that juice write on as many bits of parchment as needed these words: ‘The Lord said: Increase . + . uthihot . + . and multiply . + . thabethay . + . and fill the earth . + . amah + .’ If a man wears around his neck a slip inscribed with such juice and these very words while he has intercourse with a woman then the woman will conceive a male. And, conversely, if the woman [wears it around her neck] a female. Another remedy.” Perhaps a second remedy was intended to be added, but was never recorded. On this and other remedies for sterility in Gilbertus, see RiderCatherine, Magic and Impotence in the Middle Ages (Oxford: Oxford University Press, 2006), 163–65. St. John the Baptist, whose day is significant to this ritual, was born of a sterile mother and appears prominently in childbirth charms, as shown below.

18
See McVaughMichael, “Who Was Gilbert the Englishman?,” in The Study of Medieval Manuscripts of England: Festschrift in Honor of Richard W. Pfaff, ed. BrownGeorge HardinVoigtsLinda Ehrsam (Tempe, Ariz.: ACMRS in collaboration with Brepols, 2010), 295–324.

19
The narrative motif citing a precedent mother, namely Elizabeth the mother of John the Baptist, appears as early as the sixth century in a copy of the Pseudo-Theodorus *Additamenta* ÖnnerforsAlf, “Magische Formeln im Dienste römischer Medizin,” in Aufstieg und Niedergang der Römischen Welt. Teil II: Principat, Band 37, 157–224, ed. HaaseW (Berlin: Walter de Gruyter, 1993), 210, no. 24.

20 Oxford, Bodleian Library, MS Junius 85/86, fol. 17r.

21 “Maria uirgo peperit Christum / Elizabet sterelis peperit Johannem Baptistam. / Adiuro te infans si es masculus aut femina per patrem et filium et spiritum sanctum / ut exeas et recedas ultra ei non noceas neque insipientiam illi facias amen. / Videns Dominus flentes sorores Lazari ad monumentum lacrimatus est / coram iudeis et clamabat / Lazare, ueni foras / et prodiit ligatis manibus et pedibus qui fuerat quatriduanus mortuus / Writ ðis on wexe ðe næfre ne com to nanen wyrce and bind under hire swiðran fot.” The phrase “ut exeas” is addressed to the infant; the phrase “exeas et recedas” is a formula for exorcism to which the rest of the adjuration conforms. The exorcism associated with baptism would be appropriate here if the child’s life and soul were in jeopardy.

22
The amulet, with its narrative of holy women, Christ, and Lazarus, may be understood as a “contact relic.” See Wogan-BrowneJocelyn, “The Apple’s Message: Some Post-conquest Hagiographic Accounts of Textual Transmission,” in Late-Medieval Religious Texts and Their Transmission: Essays in Honour of A. I. Doyle, ed. MinnisA. J (Cambridge: D.S. Brewer, 1994), 39–53, esp. 43. What matters for this essay is its communicative effect on the vulnerable woman who is aware that the holy words are being brought to bear on her circumstances, her experience.

23
ElsakkersMarianne, “‘In Pain You Shall Bear Children’ (Gen. 3:16): Medieval Prayers for a Safe Delivery,” in Women and Miracle Stories: A Multidisciplinary Exploration, ed. KorteAnne-Marie (Leiden: Brill 2001), 179–209 (esp. 182–93); (FranzAdolph, Die kirchlichen Benediktionen im Mittelalter, 2 vols. (Freiburg im Breisgau: Herder, 1909), 2:198–203; OhrtFerdinand, “Gebärsegen,” Handwörterbuch des deutschen Aberglaubens (Berlin: Walter de Gruyter, 1931), 3, cols. 344–46. For the liturgical history of the stem of Jesse and its association with the Virgin, see FasslerMargot, “Mary’s Nativity, Fulbert of Chartres, and the Stirps Jesse: Liturgical Innovation circa 1000 and Its Afterlife,” Speculum 75 (2000): 389–434.

24 London, British Library, MS Royal 12.B.XII, as quoted by Hunt, *Popular Medicine* (n. 13), 133. See also British Library, Sloane MS 3550, fol. 98r, ca. 1300, 302–3. Hunt, Chap. 3 deals with the *Lettre d’Hippocrate*.

25 Whether the priest or a woman assistant attaches the amulet is not clear.

26
British Library, Sloane MS 146, fols. 30r–30v, “towards the end of the 13th cent.” Hunt, *Popular Medicine* (n. 13), quote 264, text 278; British Library, Harley MS 273, fol. 213v, fourteenth century, 90. It appears that the English cleric John Mirfield translated the Anglo-Norman instructions for this ritual into Latin in his treatise *Breviarium Bartholomei*, pars 6, dist 3, cap.xi “De difficultate partus,” British Library, Harley MS 3, fol. 128ra, ca. 1400, 90. On Mirfield, see GetzFaye, “Mirfield, John (*d*. 1407),” in Oxford Dictionary of National Biography (Oxford: Oxford University Press, 2004), http://www.oxforddnb.com/view/article/18817 (accessed 11 25, 2014).

27
British Library, Additional MS 37786, fol. 43r, first half of the fifteenth century, where the charms follow Macer’s *Herbal* See SheldonSuzanne E, “Middle English and Latin Charms, Amulets and Talismans from Vernacular Manuscripts” (Ph.D. diss., Tulane University, 1978), no. 98. British Library, Harley MS 2378, fol. 144v, and British Library, Sloane MS 521, fol. 253v, have copies of the charm for live birth. Cambridge, Cambridge University Library, MS Ee. 1. 15, fol. 78r, gives the ritual, but suggests, curiously, that bloodletting is good rather than bad for the mother to be.

28
See the discussion of remedy books in Hunt, *Popular Medicine*; VoigtsLinda Ehrsam, “Scientific and Medical Books,” in Book Production and Publishing in Britain, 1375–1475, ed. GriffithsJeremyPearsallDerek (Cambridge: Cambridge University Press, 1989), 345–402; (GetzFaye, Medicine in the English Middle Ages (Princeton, N.J.: Princeton University Press, 1998), chap. 3. See also MitchellLaura Theresa, “Cultural Uses of Magic in Fifteenth-Century England” (Ph.D. diss., University of Toronto, 2011), esp. chap. 2 on Cambridge, Trinity College, MS O.1.57, a manuscript belonging to the Haldenby family, gentry of Northamptonshire. Mitchell shows how knowledge of magical texts contributed to the formation of the family’s educational and intellectual identity.

29
OlsanLea, “The Corpus of Charms in the Middle English Leechcraft Remedy Books,” in Charms, Charmers and Charming: International Research on Verbal Magic, ed. RoperJonathan (Houndmills: Palgrave Macmillan, 2009), 214–37, ritual on 219–20. Cf. HeinrichFritz, ed., Ein Mittelenglisches Medizinbuch (Halle: M. Niemeyer, 1896), 143–44, http://archive.org/stream/einmittelenglisc00heinuoft#page/143/mode/2up/search/143 (accessed 8 2, 2014). The English manuscript dated 1475 once owned by John Brand (1744–1806) had the same ritual, and may in fact have been a member of the “Leechcraft” group. See HelmKarl, “Mittelalterliche Geburtsbenediktionen,” Hessische Blätter für Volkskunde 9 (1910): 208–11.

30 Cantusdatabase website, http://cantusdatabase.org/id/006716a (accessed August 2, 2014). Such chants originated as part of monastic practice of communal liturgical worship, or Offices, at seven times during the day (Matins, Lauds, Prime, Terce, Sext, Vespers, and Compline).

31 Elsakkers’s argument (“‘In Pain You Shall Bear Children’” [n. 23]) that part of the *peperit* charm was probably employed as “work song” by the attendant women seems unlikely given its prescribed amuletic use.

32
Lincoln, Lincoln Cathedral, MS 91, fols. 303r–304r: facsimile in BrewerD. SOwenA. E. B, “Introductions,” in The Thornton Manuscript (Lincoln Cathedral MS 91) (London: Scolar Press, 1975; 2nd ed. 1977); OgdenMargaret S, The ‘Liber de diversis medicinis’ in the Thornton manuscript (MS. Lincoln cathedral A.5.2.), Early English Text Society, o.s. 207 (London: EETS, 1938), 56–57; KeiserGeorge R, Works of Science and Information, in *A Manual of the Writings in Middle English 1050–1500*, ed. HartungAlbert E (New Haven: Connecticut Academy of Arts and Sciences, 1998), vol. 10, no. 272, 3656, 3841, describes the group. OrlemanskiJulie, “Thornton’s Remedies and the Practices of Medical Reading,” in Robert Thornton and His Books: Essays on the Lincoln and London Thornton Manuscripts, ed. FeinSusannaJohnstonMichael (York: York Medieval Press, 2014), 235–55, is the most recent study.

33 “An oþer charme. Nota bene optime. Beata Anna [g]enuit sanctam Mariam matrem Domini nostri Dei nunciante Gabriele arcangelo. Per ipsam natiuitatem credo quia omnis christianus a morte & ab omni periculo potest esse liberatus. Sancta Maria Dei genitrix & omnes apoltoli & omnes martires & omnes sancti confessores & omnes sancte virgines intercedant pro famula Dei .N. Amen.” Ogden, *Liber* (n. 32), 57.

34
L’EstrangeElizabeth, Holy Motherhood: Gender, Dynasty, and Visual Culture in the Later Middle Ages (Manchester: Manchester University Press, 2012), 59–60 and 73, n. 67. For a possible Anglo-Saxon occurrence, see OlsanLea, “The Arcus Charms and Christian Magic,” Neophilologus 73 (1989): 438–47. A list of manuscripts containing the charm appears in Keiser, Works of Science (n. 32), vol. 10, no. 349 (3874).

35
Önnerfors, “Magische Formeln” (n. 19), 210, no. 25 For continental charms, see also Franz, Die kirchlichen Benediktionen (n. 23), 2:201.

36 The earliest occurrence of this set of rituals found together we know of is in the Latin remedy book of Master John Bray or Braize, to whom is attributed a *Practica medicinae* extant in the fourteenth-century part of British Library, MS Sloane 521, fols. 128r–159v. At the end of this text is a section introduced as “Carmina ponuntur in hoc capitulo pro diversis infirmitatibus” (Charms are placed in this chapter for different ailments), charms used, Bray says, by modern authorities, though not by ancients. He does not indicate the sources for his ritual formulas. Here we find the *Arcus* (“Arthurus”), *Oceanum*, and *Quicumque vult* rituals, and also the ritual beginning *Beata Anna* (all fol. 159r).

37 Cambridge University Library, MS Dd.V.53, fol. 107r-v: “Ut mulier paret cito infantem que diu laborat. ligetur istud carmen infra genua mulieris parturientis cum oratione dominica & salutacionem beate virginis + Sicut vere credimus quod verbum + caro + factum est. + et quod maria virgo deum peperit infantem deum verum & hominem. Sic & tu ancilla maria intercedente & domino magistro Jesu christo iubente feliciter. parias prolem quam in utero habes. Quando christus natus est nullum dolorem eius mater passa est. Denuo christus deus & homo nascitur. + infans te vocat christus + exi + exi + christus vincit + christus regnat + christus_imperat + christus te ab omni malo defendat amen. + michael + gabriel + raphael + venite in adiutorium ei.” Arderne’s charm also appears in British Library, Sloane MS 56, fol. 9v and Bodleian Library, Digby MS 88, fol. 3v.

38
Manuscripts of the *Liber medicinalium* are listed in SharpeRichard, A Handlist of the Latin Writers of Great Britain and Ireland before 1540 (Turnhout: Brepols, 1997), 203 See OlsanLea T, “Charms and Prayers in Medieval Medical Theory and Practice,” Soc. Hist. Med. 16 (2003): 365; on Arderne as author and compiler, see JonesPeter Murray, “John of Arderne and the Mediterranean Tradition of Scholastic Surgery,” in Practical Medicine from Salerno to the Black Death, ed. García-BallesterLuis  (Cambridge: Cambridge University Press, 1994), 289–321.

39
British Library, Harley MS 2558; ThorndikeLynnKibrePearl, A Catalogue of Incipits of Mediaeval Scientific Writings in Latin (London: Mediaeval Academy of America, 1963), col. 1239D; TalbotC. HHammondE. A, The Medical Practitioners in Medieval England: A Biographical Register (London: Wellcome Historical Medical Library, 1965), 125; Getz, Medicine (n. 28), 106–7. See JonesPeter Murray, “Harley MS 2558: A Fifteenth-Century Medical Commonplace Book,” in Manuscript Sources of Medieval Medicine, ed. SchleissnerM. R (New York: Garland, 1995), 35–54; JonesPeter Murray, “Thomas Fayreford: An English Fifteenth-Century Medical Practitioner,” in Medicine from the Black Death to the French Disease, ed. FrenchRogerArrizabalagaJonGarcía-BallesterLuis (Aldershot: Ashgate, 1998), 156–83; and Olsan, “Charms and Prayers” (n. 38), 346–47; Oxford Dictionary of National Biography, http://www.oxforddnb.com/view/article/45762 (accessed 8 2, 2014)

40 British Library, Harley MS 2558, fol. 118v.

41
For more on *experimenta and empirica*, see Olsan, “Charms and Prayers” (n. 38), 347–57; JonesPeter MurrayOlsanLea T, “Takamiya MS 61: A Book of Experiments,” Poetica 73 (2009): 87–107.

42
WaltherHans, ed., Initia Carminum ac Versuum Medii Aevi Posterioris Latinorum (Göttingen: Vandenhoeck & Ruprecht, 1959), no. 20383.

43 Franz, *Die kirchlichen Benediktionen* (n. 23), 2:201, no. 8, 202, no. 2, published charms containing these Virgilian lines in a twelfth-century medical manuscript from Vienna and also inscribed in a thirteenth- or fourteenth-century medical collection.

44 Cf. the ritual for writing the Lazarus motif on pieces of apple for the parturient woman to consume in British Library, Royal MS 12.B.XXV, fol. 61v.

45
British Library, Harley MS 2558, fol. 117v. The same lines from the *Aeneid* are found in a childbirth amulet in the medical notebook of the English scholar William Worcester (British Library, Sloane MS 4, fol. 35r), late fifteenth century. The third amulet is found in expanded form with instructions in Middle English in New Haven, Yale Medical Historical Library, Cushing/Whitney MS 47, fol. 38r, third quarter of fifteenth century (see OlsanLea T, “The Language of Charms in a Middle English Recipe Collection,” ANQ 18 [2005]: 31–37). For a discussion of Fayreford’s prescriptions in relation to surviving examples of amulets, see JonesPeter Murray, “Amulets: Prescriptions and Surviving Objects from Late Medieval England,” in Beyond Pilgrim Souvenirs and Secular Badges: Essays in Honour of Brian Spencer, ed. BlickSarah (Oxford: Oxbow, 2007), 92–107.

46
“Ut partus facilitetur scribe istud & liga super ventrem illius. Maria peperit christum + anna mariam + elizabeth. Johannem + selina.remigium + sator + arepo + tenet + opera + rotas et bibat folium diptanni” (Cambridge, King’s College, MS 16, fol. 93v). Cf. British Library, Sloane MS 3550, fol. 98r, ca. 1300, with Anglo-Norman rubrics, as quoted in Hunt, Popular Medicine (n. 13), 302–3. On the friars’ handbook, see JonesPeter Murray, “The *Tabula Medicine*: An Evolving Encyclopaedia,” in English Manuscript Studies 1100–1700, vol. 14, *Regional Manuscripts 1200–1700*, ed. EdwardsA.S.G (Chicago: University of Chicago Press, 2008), 60–85; JonesPeter Murray, “Mediating Collective Experience: The *Tabula Medicine* as a Handbook for Medical Practice,” in Between Text and Patient: The Medical Enterprise in Medieval and Early Modern Europe, ed. GlazeFlorence ElizaNanceBrian K (Florence: Sismel-Edizioni del Galluzzo, 2011), 279–307.

47
SkemerDon C, “Amulet Rolls and Female Devotion in the Late Middle Ages,” Scriptorium 55 (2001): 197–227, esp. 202–14 and 224–27; Skemer, Binding Words (n. 2) on amulets for women, 235–78; SkemerDon C, “Written Amulets and the Medieval Book,” Scrittura e civiltà 23 (1999): 253–305.

48
FriedmanJohn B, Northern English Books, Owners, and Makers in the Late Middle Ages (Syracuse, N.Y.: Syracuse University Press, 1995), 167–70.

49
DillingW. J, “Girdles: Their Origin and Development, Particularly with Regard to Their Use as Charms in Medicine, Marriage, and Midwifery,” Caledonian Med. J. 9 (1912–14): 337–57 and 403–25 (421). See Privy Purse Expenses of Elizabeth of York, https://archive.org/stream/privypurseexpens00nicouoft#page/78/mode/2up/search/December+1502 (accessed 8 2, 2014).

50
MorseMary, “Alongside St Margaret: The Childbirth Cult of Saints Quiricus and Julitta in Late Medieval English Manuscripts,” in Manuscripts and Printed Books in Europe, 1350–1550: Packaging, Presentation and Consumption, ed. CayleyEmmaPowellSusan (Liverpool: Liverpool University Press, 2013), 187–206 (183 and n. 30); MorseMary, “Thys moche more ys oure lady mary longe’: Takamiya MS 56 and the English Birth Girdle Tradition,” in Middle English Texts in Transition: A Festschrift Dedicated to Toshiyuki Takamiya on His 70th birthday, ed. HorobinSimonMooneyLinne (York: York Medieval Press, 2014), 199–219.

51 London, Wellcome Library, MS 804 and MS 804A.

52 St. Margaret of Antioch was one of the Fourteen Holy Helpers, who were sought for healing from the fourteenth century. Having been delivered from the devil in the form of a dragon, she was the patron of safe delivery of a child. Her life appears in English as early as the eleventh century.

53
Books of Hours (whose owners were often female) were often themselves viewed as amuletic, as pointed out by Skemer, “Amulet Rolls” (n. 47), 214–17.

54 For example, Wellcome Library, MS 804A.

55
Ten are well known: London, British Library, MS Additional 88929; London, British Library, Harley Roll T 11; London, British Library, Harley Charter 43 A 14; London, Wellcome Library, MS 632; London, Wellcome Library, MS 804; London, Wellcome Library, MS 804A; New Haven, Beinecke Library, MS 410; New Haven, Beinecke Library, Takamiya MS 56; New York, Pierpoint Morgan Library, Glazier MS 39; fifteenth-century roll in a private collection described by Skemer in “Amulet Rolls” (n. 47); for STC 14547.5, a printed roll, see GwaraJoseph JMorse, “A Birth Girdle Printed by Wynkyn de Worde,” Library, 7th ser., 13 (2012): 33–62.

56 Suffering the pains of childbirth was punishment for Eve’s sin (Genesis 3:16).

57 On the back of the roll, the stated recipient of Wellcome Library, MS 632 is Pope Leo, who received the missive in a golden coffer. The inscription stipulates that whoever carries it upon him “wyth trewe fayth and good devocyon” and says five paternosters and five aves and a creed “in the worshypp of hym that thys mesure ys of” (i.e., Christ) will escape all manner of violence, not die in deadly sin, and prosper.

58 From the dorse of Wellcome Library, MS 632.

59
On such practices, see RudyKathryn M, “Kissing Images, Unfurling Rolls, Measuring Wounds, Sewing Badges and Carrying Talismans: Considering Some Harley Manuscripts through the Physical Rituals They Reveal,” Electronic Brit. Libr. J. (2011): 1–56, www.bl.uk/eblj/2011articles/pdf/ebljarticle52011.pdf (accessed 8 2, 2014).

60
For example, see the Assumption from the altarpiece by Matteo di Giovanni, dated “probably 1474,” London, National Gallery, NG 1155. On the comparative widths of prayer rolls and their significance for amuletic purposes, see EdsallMary Agnes, “*Arma Christi*` Rolls or Textual Amulets? The Narrow Roll Format Manuscripts of ‘O Vernicle,’” Magic, Ritual, and Witchcraft 9, no. 2 (2014): 178–209, esp. 199–209.

61 British Library, Harley Roll T 11.

62
On the performance of these rituals, see GarnerLori Ann, “Anglo-Saxon Charms in Performance,” Oral Tradition 19 (2004): 20–42; WestonL.M.C, “Women’s Medicine, Women’s Magic: The Old English Metrical Childbirth Charms,” Mod. Philol. 92 (1995): 279–93. Scholars have disagreed about how to divide this set of charms and rituals. For a summary, see PettitEdward, Anglo-Saxon Remedies, Charms and Prayers from British Library MS Harley 585: The Lacnunga, 2 vols. (Lewiston, N.Y.: Edwin Mellen Press, 2001).

63
Quotations are adapted from Pettit, Anglo-Saxon Remedies (n. 62), 1:112–15 Translations are freely adapted from Garner, “Anglo-Saxon Charms” (n. 62) and Pettit, Anglo-Saxon Remedies (n. 62).

64
“Se wifmon se hyr bearn afedan ne mæg: genime heo sylf hyre agenes cildes gebyrgenne dæl, [w]ry æfter þon(ne) on blace wulle 7 bebicge to cepemannu(m) 7 cweþe þon(ne): ‘Ic hit bebicge; ge hit bebicgan / Þas sweartan wulle 7 þysse sorge corn.’” Pettit, Anglo-Saxon Remedies (n. 62), 1:112.

65
“Se man se (n)e mæge bearn afedan.” Ibid., 1:114. MeaneyAudrey L, “Women, Witchcraft and Magic in Anglo-Saxon England,” in Superstition and Popular Medicine in Anglo-Saxon England, ed. ScraggDonald G (Manchester: Manchester Centre for Anglo-Saxon Studies, 1989), 9–40 (esp. 24–25), thinks that *afedan* in this instance refers to nurture after, rather than before, birth, thus to lactation. The implication in the poetic lines that the fetus is fully formed and the rituals taking liquids and nourishment lend weight to this view.

66 “nime þon(ne) anes bleos cu meoluc on hyre handæ 7 gesupe þon(ne) mid hyre muþe, 7 gange þon(ne) to yrnendu(m) wætere 7 spiwe þærin þa meolc, 7 hlade þon(ne) mid þære ylcan hand þæs wæteres muđ fulne 7 forswelge; cweþe þon(ne) þas word: ‘Gehwer ferde Ic me þone mæran maga þihtan. / mid þysse mæran mete þihtan / þon[ne] ic me wille habban 7 ham gan’. Þon(ne) heo to þan broce ga, þon(ne) ne beseo heo no, ne eft þon(ne) heo þanan go; 7 þon(ne) ga heo in oþer hus oþer heo ut ofeode, 7 þær bebyrge metes.” Pettit, *Anglo-Saxon Remedies* (n. 62), 1:114.

67
In Bald’s Leechbook LXIII, CockayneT. O, Leechdoms, Wortcunning, and Starcraft of Early England, Rolls Series, no. 35, 3 vols. (London: Longman, Green, 1864–66), 2:138, 139; Leechbook III, LXI, LXII, in 2:344–45; 346–47; Lacnunga LXIII; Pettit, Anglo-Saxon Remedies (n. 62), 1:32, 33.

68
Cambridge, Corpus Christi College, MS 41, p. 239. The manuscript can be seen online at http://parkerweb.stanford.edu/parker/actions/page_turner.do?ms_no=41 (accessed August 2, 2014). On charms and prayers added in the margins of the manuscript, see, for example, KeeferSarah Larratt, “Margin as Archive: The Liturgical Marginalia of a Manuscript of the Old English Bede,” Traditio 51 (1996): 147–77; JollyKaren Louise, “On the Margins of Orthodoxy: Devotional Formulas and Protective Prayers in Cambridge, Corpus Christi College MS 41,” in Signs on the Edge: Space, Text and Margin in Medieval Manuscripts, ed. KeeferSarah LarrattBremmerRolf HJr. (Paris: Peeters, 2007), 135–83; OlsanLea, “The Marginality of Charms in Medieval England,” in The Power of Words: Studies on Charms and Charming in Europe, ed. KapalóJamesPócsEvaRyanWilliam (Budapest: Central European Press, 2013), 135–64 (esp. 140–49); and OlsenKarin, “Thematic Affinities between the Non-liturgical Marginalia and the Old English Bede in Cambridge, Corpus Christi College 41,” in Practice in Learning: The Transfer of Encyclopaedic Knowledge in the Early Middle Ages, Mediaevalia Groningana New Series, ed. BremmerRolf HDekkerKees (Leuven: Peeters, 2010), 133–45. Olsan and Olsen reach some of the same conclusions independently.

69
Franz, Die kirchlichen Benediktionen (n. 23), 2:190 and Olsan, “Marginality” (n. 68), 143.

70
JollyKaren, “On the Margins” (n. 68), 138, raises the possibility that a feminine ending in the prayer and two other details in the marginalia of Corpus MS 41 might “suggest a lay female population possibly served by religious women functioning as midwives” as the milieu in which they were copied, “although these items could reflect just as well the source texts’ provenance rather than the marginal scribe’s context.” But further evidence for this interpretation has not emerged.

71
Cf. Franz, Die kirchlichen Benediktionen (n. 23), and evidence from ecclesiastical benedictions from Central Europe in PócsÉva, “Church Benedictions and Popular Charms in Hungary,” 165–97, and TimotinEmanuela, “The *Nˇajit* Between Prayers and Charms: A Study of the Romanian Manuscript Tradition,” 239–56, both in Kapaló, Pócs, and Ryan, *Power of Words* (n. 68).

72
BillerPeter, “Childbirth in the Middle Ages,” Hist. Today 36 (1986): 42–49, and LeeBecky, “A Company of Women *and* Men: Men’s Recollections of Childbirth in Medieval England,” J. Fam. Hist. 27 (2002): 92–100.10.1177/03631990020270020212004900

73
For example, Cambridge University Library, MS Ii.VI.2, fol. 10r; (DuffyEamon, Marking the Hours: English People and Their Prayers 1240–1570 (New Haven, Conn.: Yale University Press, 2006), 86.

